# Mammography screening in the Netherlands: delay in the diagnosis of breast cancer after breast cancer screening

**DOI:** 10.1038/sj.bjc.6602158

**Published:** 2004-10-26

**Authors:** L E M Duijm, J H Groenewoud, F H Jansen, J Fracheboud, M van Beek, H J de Koning

**Affiliations:** 1 Department of Radiology, Catharina Hospital, Michelangelolaan 2, 5623 EJ Eindhoven, The Netherlands; 2 Department of Public Health, Erasmus MC, University Medical Center Rotterdam, PO Box 1738, 3000 DR, The Netherlands; 3 Regional Laboratory for Pathology, PAMM Laboratories, Michelangelolaan 2, 5623 EJ Eindhoven, The Netherlands

**Keywords:** breast neoplasms, breast radiography, breast radiography, quality assurance, cancer screening

## Abstract

In a prospective study we determined the frequency and causes of delay in the diagnosis of breast cancer after suspicious screening mammography. We included all women aged 50–75 years who underwent biennial screening mammography in the southern breast cancer screening region of the Netherlands between 1 January 1996 and 1 January 2002. Clinical data, breast imaging reports, biopsy results and breast surgery reports were collected of all women with a positive screening result with a minimum of 2-year follow-up. Of 153 969 mammographic screening examinations, 1615 (1.05%) were positive screens. Breast cancer was diagnosed in 770 (47.9%) of 1607 women for whom follow-up information was available, yielding a cancer detection rate of 5.0 per 1000 women screened. Breast cancer was diagnosed within 3 months following a positive screen in 722 cases (93.8%). The diagnostic delay was 4–6, 7–12 and 13–24 months, respectively, in 11 (1.4%), 24 (3.1%) and nine (1.2%) patients. In four other patients (0.5%), breast cancer was diagnosed after a repeat positive screen, resulting in a diagnostic delay of 25–27 months. Reasons for a diagnostic delay >3 months were erroneous mammographic interpretation of suspicious lesions as benign or probably benign lesions (33 cases), benign biopsies from a malignant lesion (10), and omission to biopsy or remove a lesion that was suspicious at breast imaging (4) or core biopsy (1). We conclude that there is room for improvement in the workup of patients with a positive screening mammography, as seen from data in this screening region. To improve the workup, we suggest that other breast cancer screening programmes also identify delay in breast cancer diagnosis after a positive screen.

Breast cancer is the most frequent malignancy among women in developed countries (Parkin *et al*, 1997; Parkin, 2001). Breast screening aims to detect breast cancers as small as possible and before lymph node dissemination. Randomised trials of mammographic screening have provided strong evidence that early diagnosis and treatment of breast cancer reduce breast cancer mortality (Nystrom *et al*, 2002).

In the Netherlands, a nation-wide breast cancer screening programme was gradually implemented from 1989 till 1997 (Fracheboud *et al*, 2001). The programme initially offered biennial screening mammography to women aged 50–69 years; in 1998 the upper age limit was extended to 75 years. The overall attendance rate is 80%; today, about 800 000 screening examinations per year are performed at 63 fixed or mobile screening units.

Screening does not end at the early recognition of mammographic abnormalities that are suspect for breast cancer: a prompt diagnostic follow-up is important as well. Delay in the diagnosis of breast cancer, however, frequently occurs. In the United States, physician delay is the most common cause of malpractice (Kern, 1994; Physician Insurers Association of America, 2003), often involving the inappropriate reassurance that a palpable mass is benign without having performed biopsy (Goodson and Moore, 2002). In mammography screening programmes, mammographic abnormalities usually are nonpalpable. To our knowledge, no prospective data of the frequency and the reasons of diagnostic delay of non-palpable breast cancers are available.

The aim of this observational follow-up study was to determine the frequency and causes of delay in the diagnosis of breast cancer after suspicious mammographic findings at screening.

## MATERIALS AND METHODS

### Study population and screening procedure

We included all women who underwent screening mammography at one of two specialised screening units (one fixed and one mobile) in the southern breast cancer screening region of the Netherlands (BOBZ, Bevolkings Onderzoek Borstkanker Zuid) between 1 January 1996 and 1 January 2002. Details of the nation-wide breast cancer screening programme have been described previously (Fracheboud *et al*, 1998; Duijm *et al*, 2004). In brief, women aged 50–75 years are invited by letter every 2 years to attend breast screening. In initial screens, that is, the first time women are screened within the screening programme, two-view (medio-lateral-oblique and cranio-caudal) mammography of each breast is performed. In subsequent screens one-view mammography is standard, though two-view mammography is obtained in 20–30% of subsequent screens; the indications include complicated judgement due to breast surgery or dense fibroglandular tissue; any changes in mammographic findings; and a longer-than-2-year interval since the previous screen. All examinations are read independently by two certified screening radiologists. Women with normal or benign mammographic findings or with nonspecific minimal signs are not referred for further diagnostic workup (Maes *et al*, 1997). In case of suspicious or malignant lesions, the general practitioner (GP) refers the woman to a surgical oncologist in a regional hospital. After physical examination by the surgeon, a two-view mammogram of each breast is obtained; local compression or magnification mammograms are performed if necessary. Depending on the diagnostic workup protocols and the facilities available, further evaluation may also include breast ultrasonography, magnetic resonance mammography, fine needle aspiration biopsy (FNAB, cytology) or histologic core biopsy or excision biopsy (Kwaliteitsinstituut voor de Gezondheidszorg CBO, 2000). The 1999 Dutch national guidelines for the early detection and diagnosis of breast cancer recommend that the diagnostic process should be completed within 4 weeks in at least 90% of patients to minimise the period of uncertainty. In our study, we defined a longer-than-3-month interval between the positive breast screening and the confirmation of breast cancer as diagnostic delay.

### Screening follow-up

We collected data on diagnostic procedures, breast cancer diagnosis, histopathology and TNM-classification for all women with a positive screening. The follow-up of women with a positive screening included the period through the next screening round (with a screening interval of about 2 years). In total, 13 women died during follow-up; linkage with the pathology laboratories showed that none of these women had suffered from breast cancer. Following standard procedures, the GP informed the BOBZ screening organisation to which hospital the woman had been referred. At 3–6 months after the referral, the BOBZ collected the copies of radiology reports and of surgical records for each referred woman. In addition, breast pathology reports were obtained from the regional pathology laboratories, and radiotherapy reports from the regional radiotherapy institute. To trace breast cancers diagnosed more than 6 months after referral, information in addition to the standard follow-up procedure was sought: (a) inquiry about pathology specimens of women with a repeat positive screen for the same lesion that had been considered benign at workup 2 years before; (b) linkage with the regional pathology laboratories of women who had not re-attended screening 2 years after a false positive screening (mammographic screening was defined as false-positive if further diagnostics after referral proved to be negative); and (c) linkage to the regional Register of Deaths (Gemeentelijke Basisadministratie Persoonsgegevens) 2 years after a false-positive screen.

For cases with bilateral disease, the cancer with the highest stage was retained and multiple foci of cancer in one breast were counted as one cancer.

To determine whether a delay in breast cancer diagnosis could be attributed to the radiological assessment, two dedicated breast radiologists (LD, FJ) independently and retrospectively reviewed the diagnostic breast images of all women with diagnostic delay. Each reviewer classified the lesions using the Breast Imaging Reporting and Data System BI-RADS (American College of Radiology, 1998). For BI-RADS three lesions (nonpalpable probably benign lesions), the criteria published by Sickles (1991) and by Varas *et al* (2002) were used.

### Workup facilities

One of the authors (LD) inquired at the hospitals involved in the diagnostic workup whether an outpatient breast clinic was present; whether sophisticated diagnostic modalities such as magnetic resonance mammography and stereotactic core biopsy were available; whether the follow-up results of women with a positive screen were regularly discussed by a multidisciplinary team of clinicians, radiologists and pathologists; and whether the hospital radiologists also participated in the nation-wide breast cancer screening programme.

All women included in our study had given written informed consent to use their data for scientific purposes before participation in the screening programme. Institutional review board approval was not required for this type of study.

## RESULTS

### Referral and diagnostic follow-up examinations

A total of 153 969 mammographic screening examinations were performed: 41 683 were initial screens and 112 286 were subsequent screens. The mean age of the screened women was 58 years (range 50–75 years). In total, 1615 women were referred for further diagnostic examination (Table 1

**Table 1:** Referral and diagnostic follow-up for initial and subsequent screens between 1 January 1996 and 1 January 2002

			**Subsequent screens**							
	**Initial screens**		**Interval <30 mo**		**Interval ⩾30 mo**		**All subsequent screens**		**Total**	
	***N*=41 683**		***N*=106 035**		***N*=6251**		***N*=112 286**		***N*=153 969**	
Referrals (*N*)	592		914		109		1023		1615	
Referral rate (per 1000 women screened)	14.2		8.6		17.4		9.1		10.5	
										
	***N***	**%**	***N***	**%**	***N***	**%**	***N***	**%**	***N***	**%**
Diagnostic follow-up available^a^	587	99.2	911	99.7	109	100.0	1020	99.7	1607	99.5
*Diagnostic procedures*^b^										
Breast imaging	183	31.2	330	36.2	25	22.9	355	34.8	538	33.5
FNAB (cytology)	18	3.1	44	4.8	8	7.3	52	5.1	70	4.4
Surgical or core biopsy	386	65.8	537	58.9	76	69.7	613	60.1	999	62.2
										
*Outcome*										
Breast cancer^c^	273	46.5	431	47.3	66	60.6	497	48.7	770	47.9
No breast cancer	314	53.5	480	52.7	43	39.4	523	51.3	837	52.1
										
Biopsy rate (per 1000 women screened)	9.7		5.5		13.4		5.9		6.9	
Detection rate (per 1000 women screened)	6.5		4.1		10.6		4.4		5.0	
Positive predictive value of referral (%)	46.5		47.3		60.6		48.7		47.9	
Positive predictive value of biopsy (%)^d^	67.6		74.2		78.6		74.7		72.0	

a Diagnostic follow-up was missing for eight women: five patients refused any kind of workup after a positive screen; workup remained undone in one patient who also had metastasised lung cancer; the general practitioners of two other women reported that the screen-detected lesion had remained unchanged for several years and that referral therefore was not indicated.

b The diagnostic procedures were classified according to the most invasive diagnostic technique. In all instances, the women underwent clinical breast examination as well. The women who underwent FNAB or histological (surgical or core) biopsy also had had breast imaging.

c Of 770 breast cancers, 48 were diagnosed more than 3 months after the positive mammographic screening.

d The eight cases without follow-up were excluded from these analyses.

). The mammographic features at screening were density (75.9%), microcalcifications (14.6%), density with microcalcifications (6.4%), asymmetry of breast parenchyma (1.3%) or architectural distortion (1.8%). Diagnostic workup was performed in 1607 women (99.5%); five other women refused further assessment; workup was refrained from in one woman with metastasised lung cancer; and the GPs of two women reported that the mammographic lesion had remained unchanged for several years and that workup was not indicated. The period between the date of the positive mammogram and the first hospital visit was less than 3 weeks in all but two women: one woman could be contacted only 6 weeks after the screening examination, the other woman agreed with further workup 2 months following the positive screen. Of the women referred at first screens, 68.2% underwent biopsy, and 65.0% of the women referred at subsequent screens; the biopsy rates (percutaneous or open surgical) were 9.7 and 5.9 per 1000 screened women, respectively.

### Breast cancer diagnosis

In total, 770 histologically proven breast cancers were diagnosed, yielding an overall cancer detection rate of 5.0 per 1000 women screened and a true-positive referral rate of 47.9% (Table 1). Breast cancer was diagnosed within 3 months following a positive screen in 722 women (93.8%). The diagnostic delay exceeded 3 months in 48 women (6.2%); 19 cases involved initial screens and 29 cases involved subsequent screens (screening interval <30 months: 26 women; screening interval ⩾30 months: three women). The delay was 4–6 months in 11 women (1.4%), 7–12 months in 24 women (3.1%) and 13–24 months in nine women (1.2%); all these women had remained under physical and radiological surveillance after the initial assessment. In four other women (0.5%), breast cancer was diagnosed after referral in the next screening round for the same lesion that had been considered benign at workup 2 years before; in these cases, the diagnostic delay was 25–27 months. The tumour size distribution and axillary lymph node status are presented in Table 2

**Table 2:** Tumour size distribution and lymph node metastases by diagnostic delay

	**Diagnostic delay**							
	**⩽3 mo (*N*=722)**		**>3 mo (*N*=48)**		**Total (*N*=770)**		**National figures (*N*=17 084)**^a^	
	**No.**	**%**	**No.**	**%**	**No.**	**%**	**No.**	**%**
*Type of breast cancer*								
DCIS	112	15.5	11	22.9	123	16.0	2457	14.6
Invasive	610	84.5	37	77.1	647	84.0	14 352	85.4
*T1a/b/c*	483	79.2	31	83.8	514	79.4		76.1
*T2*+	125	20.5	6	16.2	131	20.2		21.9
*Unknown*	2	0.3	0	0.0	2	0.3		2.0
								
*Lymph-node status of invasive cancers*								
Positive^b^	183	30.0	9	24.3	192	29.7		26.0
Negative	409	67.0	28	75.7	437	67.5		67.9
Unknown	18	3.0	0	0	18	2.8		6.0

a Unpublished data. For 275 breast cancers, it was not known whether they were DCIS or invasive cancers. *Source*: NETB (2001). Figures for screen-detected breast cancers, 1996–2001.

b Including cases with distant metastases.

. In 11 of the 48 women with a diagnostic delay ductal cancer *in situ* (DCIS) was diagnosed (22.9%); six women (12.5%) had T2+-tumours (>20 mm), and nine women (18.8%) had axillary lymph node metastases. For cancers diagnosed within 3 months following a positive screen, the percentages of DCIS, T2+-tumours and lymph node metastases were 15.5, 20.5 and 25.3%, respectively.

### Delay in breast cancer diagnosis

The reasons that contributed to a diagnostic delay are listed in Table 3

**Table 3:** Characteristics of delay of breast cancer diagnosis (48 cases with a longer-than-3-month delay)

**Characteristics of diagnostic delay**	**No.**	**%**
Nonpalpable lesion classified probably benign (BI-RADS 3) at workup, but ‘suspicious’ or ‘malignant’ in retrospect	26	54.2
Lesion classified ‘benign’ (BI-RADS 2) at workup, but ‘suspicious’ or ‘malignant’ in retrospect	6	12.5
Lesion classified ‘normal’ (BI-RADS 1) at workup, but ‘suspicious’ (BI-RADS 4) or ‘malignant’ (BI-RADS 5) in retrospect	1	2.1
*Benign open (surgical) biopsy from malignant lesion*	5	10.4
Biopsy after radiological wire localization	4	8.3
No wire localization	1	2.1
		
*Benign percutaneous biopsy from malignant lesion*	5	10.4
Fine needle aspiration biopsy	3	6.2
Core biopsy	2	4.2
		
Surgical oncologist did not follow the radiologist's advise to biopsy a radiologically suspicious lesion	4	8.3
Surgical oncologist did not follow the pathologist's advise to excise a probably malignant lesion at core biopsy	1	2.1

. In total, 33 cancers that had been classified as BI-RADS 1–3 mammograms at workup were categorised as BI-RADS 4 or 5 lesions on review: the mammographic features were density in 24 cases (72.7%), clustered calcifications in seven cases (21.2%), density with microcalcifications in one case (3.0%) and architectural distortion in one case (3.0%). In 10 patients the biopsy result proved to have been false-negative; the surgeon had not repeated biopsy despite the radiologist's explicit advice in six women, whereas clinical oncologists had followed four other women clinically despite suspicious mammograms needing further evaluation by biopsy. In one other case the surgeon did not remove a lesion after a probably malignant core biopsy result. During the 6-year inclusion period, the proportion of screen-detected cancers with diagnostic delay varied: this proportion was 9.5% (15 out of 158) in 1996; 4.7% (five out of 107) in 1997; 6.0% (seven out of 116) in 1998; 4.2% (five out of 120) in 1999; 6.7% (10 out of 149) in 2000; and 5.0% (six out of 120) in 2001.

### Screening workup facilities

At the beginning of our study in 1996, none of the eight hospitals involved in the workup of a positive screening mammogram had an outpatient breast clinic; in early 2004 this facility was available in all but one hospital. In 1998, magnetic resonance mammography had been introduced in one hospital; this modality is currently available at five sites. Stereotactic core biopsy of microcalcifications and of densities was implemented in three hospitals between 1996 and 2002 and is now routine practice at all sites. Regular discussion of positive screens by a multidisciplinary team had been established in four hospitals between 2001 and 2003; there is no such multidisciplinary approach at the other four sites. Screening radiologists are involved in the workup of screened patients at three hospitals.

## DISCUSSION

In our series, the delay in breast cancer diagnosis after a positive screening mammogram exceeded 3 months in 6.2% of the referred women, and 6 months in 4.8%. Delay in the diagnosis of breast cancer is not uncommon. Tartter *et al* (1999) found that 8% of patients in a New York Breast Service had a diagnostic delay of over 3 months from their first consultation for the breast problem that was eventually proven to result from a malignancy. In three other series, including both symptomatic and asymptomatic women, the delay exceeded 3 months in 4–39% (Jenner *et al*, 2000; Montella *et al*, 2001; Goodson and Moore, 2002). Barber *et al* (2004) recently described that 1.4% of symptomatic patients in a British breast clinic experienced a delay exceeding 2 months.

The tumour stages of breast cancers with a delayed diagnosis in our study were more favourable compared with cancers diagnosed within 3 months of a positive screen. In contrast, a Canadian study showed that screen-detected breast cancers diagnosed between 20 and 52 weeks after mammographic screening more often had a larger tumour size or lymph node metastases compared with breast cancers diagnosed within 4–12 weeks of an abnormal screen (Olivotto *et al*, 2002). Our findings may be explained by diagnostic suspicion bias: the workup may have been more aggressive for mammographic features highly suggestive of cancer.

There are conflicting reports whether diagnostic delay is associated with lower survival. Afzelius *et al* (1994) concluded that physician delay of more than 60 days was not associated with an unfavourable outcome. A systematic review, however, showed that a delay of 3–6 months in symptomatic patients was associated with lower survival (Richards *et al*, 1999). It is unknown if this adverse effect also applies to screen-detected (asymptomatic) breast cancers. An accurate and timely diagnosis, however, will minimise patient anxiety.

In our series, two-thirds of the diagnostic delay resulted from an incorrect classification of the diagnostic mammography, most frequently the erroneous classification into the BI-RADS 3 category of suspicious mammograms. Barber *et al* (2004) also found that misinterpretation of mammographic lesions as benign was one of the most common reasons for diagnostic delay. In an American study, malignant lesions for which short-term mammographic follow-up was recommended often did not fulfil the criteria for probably benign lesions in retrospect (Rosen *et al*, 2002). Other studies have consistently found that no more than 1–2% of lesions characterised as ‘probably benign’ actually turn out to be cancerous (Vizcaíno *et al*, 2001; Yasmeen *et al*, 2003).

Other reasons for the diagnostic delay in our series were false negative biopsy results or disregard of the radiologist's advise to biopsy, a radiologically suspicious lesion. Special breast care units and multidisciplinary teams may improve the assessment of symptomatic breast disease (Purushotham *et al*, 2001; Haward *et al*, 2003). In our country, breast cancer screening must be performed by certified facilities and certified screening radiologists; the workup of a positive screen, however, can take place in any hospital. Only a quarter of the hospitals in our study had outpatient breast clinics or held multidisciplinary meetings. Involving screening radiologists in the workup may be important as Dutch residents in radiology and general radiologists do not need specific knowledge of breast cancer screening. The frequency of diagnostic delay exceeding 3 months did not tend to decline during the study period. Whether delay in breast cancer diagnosis is correlated with the workup facilities at the hospitals, which since have improved, remains to be seen. The national guidelines for the early detection and diagnosis of breast cancer, published in 1999, may have influenced the course of the diagnostic process in later years as well.

We conclude that the workup of patients with a positive screening mammogram needs improvement. Services within hospitals need to be organised in order to prevent unnecessary diagnostic delay. To improve the workup after recall, we suggest that other breast cancer screening programmes also identify women with a delay in breast cancer diagnosis after a positive screen.
